# Near-infrared spectroscopy predicts events in men and women: Results from the Lipid Rich Plaque study

**DOI:** 10.1016/j.ijcha.2022.100985

**Published:** 2022-03-08

**Authors:** Frans B. Mensink, Tim J.F. ten Cate, Sander A.J. Damen, Kit Roes, Carlo Di Mario, Varinder Singh, Ziad A. Ali, William Skinner, Andre Artis, Rebecca Torguson, Cheng Zhang, Gheorghe Doros, Hector M. Garcia-Garcia, Gary S. Mintz, Robert-Jan van Geuns, Ron Waksman

**Affiliations:** a Department of Cardiology, Radboud University Medical Center, Nijmegen, the Netherlands; b Structural Interventional Cardiology, Department of Clinical & Experimental Medicine, Careggi University Hospital, Florence, Italy; c Department of Cardiology, Northwell Health, New York, NY, USA; d DeMatteis Cardiovascular Institute, St. Francis Hospital & Heart Center, Roslyn, NY, USA; e Clinical Trials Center, Cardiovascular Research Foundation, New York, NY, USA; f Department of Cardiology, Central Baptist Hospital, Lexington, KY, USA; g Department of Cardiology, Methodist Hospital, Gary, IN, USA; h The Zena and Michael A. Wiener Cardiovascular Institute, Icahn School of Medicine at Mount Sinai, New York, NY, USA; i Section of Interventional Cardiology, MedStar Washington Hospital Center, Washington, DC, USA; j Biostatistics Department, Boston University, Boston, MA, USA

**Keywords:** Near-infrared spectroscopy, Intravascular ultrasound, Sex, Non-culprit major adverse cardiac events, Lipid-rich plaque

## Abstract

**Background:**

The Lipid Rich Plaque (LRP) study demonstrated that near-infrared spectroscopy imaging of non-obstructive lesions identified patients and segments at higher risk for subsequent non-culprit major adverse cardiac events (NC-MACE). Whether this is true for both men and women is not known. In this *post hoc* analysis of the LRP study, we sought to investigate whether the maximum 4-mm Lipid Core Burden Index (maxLCBI_4mm_) was of similar predictive value in men and women for NC-MACE.

**Methods:**

Patients with an evaluable maxLCBI_4mm_ were stratified on the basis of sex at birth. A Cox proportional-hazards model was used to assess the predictive value of maxLCBI_4mm_ on future NC-MACE at the patient and plaque levels. The primary endpoint was cumulative incidence of NC-MACE at 24 months.

**Results:**

Among 1271 patients, 388 (30.5%) were women. Women were older and had a higher cardiovascular risk profile. Cumulative incidence of NC-MACE at 24 months was 10.3% for women and 7.6% for men (log-rank p = 0.11). When comparing maxLCBI_4mm_ > 400 to maxLCBI_4mm_ ≤ 400, the hazard ratio (HR) for future NC-MACE was not significantly different between sexes: 2.10 (95% confidence interval [CI]: 1.28–3.44; p = 0.003) for men and 2.24 (95% CI: 1.18–4.28; p = 0.014) for women (p = 0.87). At the plaque level, the HR comparing maxLCBI_4mm_ > 400 to maxLCBI_4mm_ ≤ 400 was 3.49 (95% CI: 1.60–7.60, p = 0.002) for men and 4.79 (95% CI: 2.02–11.38, p < 0.001) for women, which was not significantly different (p = 0.57).

**Conclusions:**

The maxLCBI_4mm_ was of similar predictive value for NC-MACE within 24 months in men and women.

## 1 Introduction

Research on atherosclerotic cardiovascular disease has been historically focused on the male patient. However, it is now well-appreciated that there can be distinct differences between men and women in various aspects of this disease, including the pathophysiology, cardiovascular risk factors, prevalence, symptom presentation, diagnosis, and treatment [1], [2]. Moreover, it has been reported that women with obstructive coronary artery disease (CAD) might have a worse prognosis than do men after myocardial infarction (MI) [3], [4]. One reason for the disparity in adverse outcomes between men and women could be a difference in the phenotype of atherosclerotic plaques. Imaging studies have investigated whether plaque composition differs between men and women, but the evidence is conflicting [5], [6], [7], [8], [9], [10]. Thus, prospective research on potential sex differences in plaque composition and its association with cardiovascular outcome is needed.

Recently, the Lipid Rich Plaque (LRP) study investigated the relationship between the presence of lipid-rich plaque detected by near-infrared spectroscopy (NIRS) and intravascular ultrasound (IVUS) imaging at unstented sites and the occurrence of subsequent major adverse cardiac events (MACE). The LRP study showed that the presence of a high maxLCBI_4mm_ (maximum Lipid Core Burden Index within a 4-mm segment) was an independent predictor of future non-culprit (NC) MACE [11], similar to other earlier studies [12], [13], [14], [15]. However, it is currently not fully elucidated whether men and women with CAD have comparable lipid content in their coronary plaques and whether the maxLCBI_4mm_ predicts future NC-MACE equally for both women and men. In this *post hoc* analysis of the LRP study, we investigated the potential differences between sexes in lipid core content at baseline as measured by NIRS-IVUS and whether it had the same diagnostic capabilities in both men and women to detect vulnerable patients and plaques. We hypothesize that the maxLCBI_4mm_ as measured by NIRS-IVUS can predict future NC-MACE similarly for women and for men.

## 2 Methods

The details of the LRP study design, methods, and endpoint analysis have been previously described [15]. For this sub-analysis, 1271 patients within whom patient-reported sex at birth and follow-up were available and for whom it was feasible to perform NIRS-IVUS of at least 50 mm of additional non-culprit territories analyzable by the core laboratory were included. All patients provided informed consent before catheterization, and the study was approved by the institutional review boards of all participating centers. Investigators were blinded to the NIRS-IVUS images. Based on the NIRS-IVUS analysis, patients were considered eligible for follow-up if they had interpretable NC segment NIRS-IVUS data, excluding by randomization 50% of the patients having plaques with maxLCBI_4mm_ < 250 as was pre-specified in the protocol. In concordance with previous studies, the LRP study used a maxLCBI_4mm_ cutoff point of 400 for the prediction of subsequent events [14], [16], [17], [18].

### 2.1 Endpoints

During the 2-year follow-up, all MACE were adjudicated by the independent clinical events committee (CEC). MACE was defined as a composite of cardiac death, cardiac arrest, non-fatal MI, acute coronary syndrome (ACS), revascularization by coronary artery bypass grafting or percutaneous coronary intervention (PCI), and rehospitalization for angina with > 20% diameter stenosis progression related and unrelated to the treatment at index procedure. If the follow-up culprit event location was identifiable by imaging or autopsy reports and this location was scanned at index with NIRS-IVUS, then the event was adjudicated for the plaque-level endpoint by the CEC masked to the baseline NIRS-IVUS data.

### 2.2 Core laboratory analysis

All NIRS-IVUS analyses were done offline by an independent core laboratory (MedStar Cardiovascular Research Network, Washington, DC, USA) using validated NIRS-IVUS analysis software (QIVUS version 3.0.16.0, Medis Medical Imaging Systems, Leiden, Netherlands). Each coronary artery was divided into 30-mm segments (referred to as Ware segments), beginning from the proximal region or ostium of the artery for plaque-level analysis, and each segment was analyzed every 1 mm. Each patient, thus, had multiple Ware segments, and the maxLCBI_4mm_ in each separate Ware segment was included for the plaque-level analysis. At the site of maxLCBI_4mm_ within each Ware segment, minimum lumen area (MLA), plaque area, plaque volume, and plaque burden (PB) were also measured.

### 2.3 Statistical analysis

In this *post hoc* analysis of the LRP study results, we stratified patients with an evaluable maxLCBI_4mm_ at baseline and complete follow-up data (24 months) by sex at birth. Descriptive statistics were used to provide the baseline characteristics with p-values for the difference. Baseline plaque characteristics were corrected for body surface area (BSA). The cumulative event rate of the primary endpoint (NC-MACE) was estimated using the Kaplan-Meier method for both sexes, and a log-rank test was used to compare the Kaplan-Meier curves. Cox proportional-hazards models were used to analyze the association of maxLCBI_4mm_ with future NC-MACE at the patient and plaque levels. At the patient level, the hazard ratio (HR) for the maxLCBI_4mm_ as a binary (operationalized as > 400 vs. ≤ 400) variable was estimated with sex as a covariate; and the interaction between sexes and maxLCBI_4mm_ was assessed. The HR for NC-MACE for a change of 100 in maxLCBI_4mm_ was estimated using the same method, and the interaction between sex and maxLCBI_4mm_ as a continuous variable was also assessed. The models were adjusted for other covariates pre-specified in the statistical analysis plan of the parent study: age, diabetes mellitus, hypertension, chronic renal insufficiency, prior smoking history, prior PCI, and presentation with an ACS.

The same analyses were used for the calculation of the association of maxLCBI_4mm_ as binary (operationalized as > 400 vs. ≤ 400) and as continuous variable at the plaque level and time to NC-MACE. For the plaque-level endpoint, the association between maxLCBI_4mm_ at a Ware segment (30 mm of coronary artery) and the occurrence of NC-MACE at the same segment during 24 months was tested. The interaction between sex and maxLCBI_4mm_ was assessed. To assess whether NIRS-IVUS can independently identify lipid-rich plaques at risk for future events, we used a frailty Cox model adjusting for high PB (>70%) and small MLA (≤4 mm^2^) as measured by IVUS. Analyses were done in SAS 9.4 (SAS Institute, Cary, NC, USA).

## 3 Results

### 3.1 Baseline measurements

Of the 1271 patients who had an evaluable maxLCBI_4mm_ and were followed for events, 388 (30.5%) were female, and 883 (69.5%) were male. The mean follow-up time was 692 (standard deviation [SD] 129) days. The baseline characteristics of the study cohort are shown in Table 1. Women tended to be older and have more cardiovascular risk factors and comorbidities. Overall, 87.5% of patients underwent PCI at the time of enrollment, with no difference between sexes.

**Table 1:** Baseline characteristics and clinical presentation.

**Characteristic**		Women (n = 388)*	Men (n = 883)*	**P-value**
Age (years)		65.6 (10.0); n = 388	63.3 (10.3); n = 882	<0.001
Age > 65 years		216/388 (55.7%)	398/882 (45.1%)	<0.001
Diabetes mellitus		166/387 (42.9%)	298/879 (33.9%)	0.002
Hypertension		339/387 (87.6%)	680/880 (77.3%)	<0.001
Peripheral vascular disease		38/382 (9.9%)	78/863 (9.0%)	0.611
Smoking history (any)		180/380 (47.4%)	507/869 (58.3%)	<0.001
Current smoker		79/380 (20.8%)	203/869 (23.4%)	0.317
History of CAD		212/351 (60.4%)	425/784 (54.2%)	0.052
Previous stroke or TIA		42/385 (10.9%)	64/875 (7.3%)	0.034
Congestive heart failure		46/383 (12.0%)	58/880 (6.6%)	0.001
Dyslipidemia		316/386 (81.9%)	697/875 (79.7%)	0.363
Chronic renal insufficiency		31/385 (8.1%)	70/882 (7.9%)	0.944
Prior PCI		151/387 (39%)	418/880 (47.5%)	0.005
Prior CABG		0/388 (0.0%)	0/880 (0.0%)	–
Prior myocardial infarction		64/378 (16.9%)	230/875 (26.3%)	<0.001
Body mass index, kg/m^2^		31.5 (8.1); n = 386	29.6 (5.6); n = 876	<0.001
Body surface area, m^2^		1.9 (0.3)	2.1 (0.2)	<0.001
Clinical Presentation at Enrollment				
Stabilized STEMI		11/388 (2.8%)	21/883 (2.4%)	0.599
Non-STEMI		205/388 (52.9%)	445/883 (50.4%)	
Stable Angina		172/388 (44.3%)	417/883 (47.2%)	
Cholesterol panel†				
	Total cholesterol, mg/dl	182.4 (49.3); n = 265	160.9 (45.4); n = 610	<0.001
	LDL, mg/dl	105.4 (44.5); n = 256	91.5 (41.0); n = 590	<0.001
	HDL, mg/dl	51.6 (17.5); n = 261	41.7 (13.9); n = 606	<0.001
	Non-HDL, mg/dl	125.7 (46.2); n = 261	115.4 (42.2); n = 604	0.001
	Triglycerides, mg/dl	146.4 (104.0); n = 260	154.8 (110.8); n = 599	0.888

Data are mean (SD) or n/N (%) unless otherwise specified. CABG, coronary artery bypass grafting; CAD, coronary artery disease; HDL, high-density lipoprotein; LDL, low-density lipoprotein; PCI, percutaneous coronary intervention; SD: standard deviation; STEMI, ST-elevation myocardial infarction; TIA, transient ischemic attack.

* Sex at birth: Women 388/1271 (30.5%); Men 883/1271 (69.5%).

† Aggregated (baseline cholesterol values or first cholesterol values within 2 months if patient was on statin therapy at enrollment).

At the patient level, there was no significant difference in maxLCBI_4mm_ between men and women (maxLCBI_4mm_ 357.33 [SD 173.42] vs. 363.41 [SD 179.13], respectively; p = 0.57). In comparison to men, there was a trend toward a higher percentage of women having a maxLCBI_4mm_ > 400 (42.5% vs. 37.1%, respectively; p = 0.07). At the plaque level, maxLCBI_4mm_ was greater in women than in men (maxLCBI_4mm_ was 174.01 [SD 181.67] vs. 161.37 [SD 175.18], respectively; p = 0.015) with a higher percentage of women having a plaque-level maxLCBI_4mm_ > 400 (14% vs. 10.6%, respectively; p < 0.001).

When corrected for BSA, men had a larger plaque area and plaque volume than women, as well as a larger PB (p < 0.001). The IVUS-derived plaque measurements are presented in Table 2.

**Table 2:** Core laboratory data.

		**Women (n = 388)**	**Men (n = 833)**	**P-value**
	**Patient-level values (n = 1271)**			
	Patient-level maxLCBI_4mm_	363.4 (179.1); n = 388	357.3 (173.4); n = 833	0.574
	Patient-level maxLCBI_4mm_ > 400	165/388 (42.5%)	165/833 (37.1%)	0.070
Ware segment values (n = 5755)*		**Women (n = 1637)**	**Men (n = 4118)**	
	Ware segment maxLCBI_4mm_	174.0 (181.7)	161.4 (175.2)	0.015
	Ware segment maxLCBI_4mm_ > 400	229/1637 (14.0%)	435/4118 (10.6%)	<0.001
*IVUS measurements*†				
	External elastic membrane_,_ mm^3^	47.8 (24.7)	53.9 (28.3)	<0.001
	Lumen area, mm^2^	7.3 (4.1)	8.0 (4.3)	<0.001
	Lumen volume, mm^3^	28.8 (16.2)	31.4 (17.3)	<0.001
	Plaque area, mm^2^	4.8 (3.1)	5.6 (3.7)	<0.001
	Plaque volume, mm^3^	19.0 (12.3)	22.5 (15.1)	<0.001
	Minimum lumen area, mm^2^	6.2 (3.6)	6.7 (3.8)	<0.001
	Minimum lumen area ≤ 4 mm	504/1631 (30.9%)	1046/4112 (25.4%)	<0.001
	Plaque burden %	37.9 (13.8)	39.3 (14.0)	<0.001
	Plaque burden ≥ 70%	15/1631 (0.9%)	44/4112 (1.1%)	0.610
	Plaque burden % at minimum lumen area	42.6 (16.0)	44.4 (15.9)	<0.001
	Plaque burden ≥ 70% at minimum lumen area	52/1631 (3.2%)	184/4112 (4.5%)	0.027
*IVUS measurements corrected for BSA*				
	External elastic lamina/BSA	25.1 (12.9)	25.6 (13.5)	0.265
	Lumen area/BSA	3.8 (2.1)	3.8 (2.1)	0.282
	Lumen volume/BSA	15.2 (8.5)	14.9 (8.3)	0.261
	Plaque area/BSA	2.5 (1.6)	2.7 (1.8)	<0.001
	Plaque volume/BSA	10.0 (6.5)	10.7 (7.2)	<0.001
	Minimum lumen area/BSA	3.3 (1.9)	3.2 (1.8)	0.24

Data are n/N (%) or mean (SD) unless otherwise specified. BSA, body surface area; MaxLCBI_4mm_, maximum 4-mm Lipid Core Burden Index; IVUS, intravascular ultrasound.

* Ware segment analysis restricted to only include segments with an evaluable maxLCBI_4mm_ value.

† IVUS measurements at the site of maxLCBI_4mm_: women (n = 1631); men (n = 4112). All IVUS measurements are within the maxLCBI_4mm_ segment only.

### 3.2 Endpoints at the patient level

**Fig. 1:**
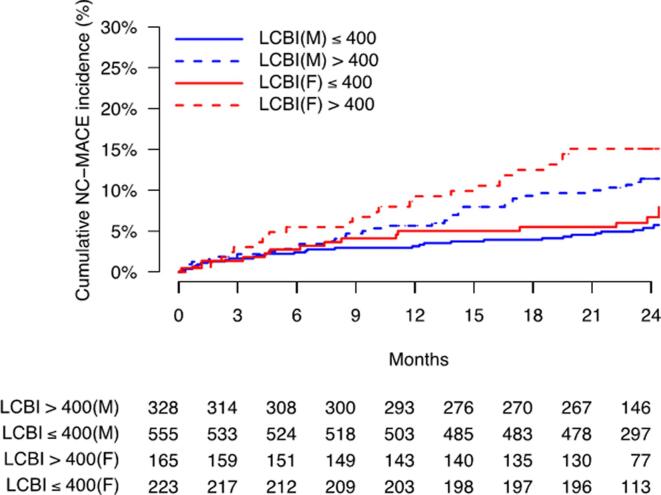
Kaplan-Meier curves of the estimated cumulative incidences of NC-MACE in men and women with maxLCBI_4mm_ > 400 vs. maxLCBI_4mm_ ≤ 400. In men, the cumulative incidence of NC-MACE in the LCBI > 400 group was 11.4% vs. 5.4% in the LCBI ≤ 400 group (log-rank test p = 0.003). In women, the cumulative incidence of NC-MACE in the LCBI > 400 group was 15.1% vs. 11.4% in the LCBI ≤ 400 group (log-rank test p = 0.012). LCBI, Lipid Core Burden Index; NC-MACE, non-culprit major adverse cardiac events.

The overall cumulative incidence of all MACE and NC-MACE at 24 months as estimated by the Kaplan-Meier method tended to be higher in women than in men (18.0% vs. 17.1% for all MACE and 10.3% vs. 7.6% for NC-MACE); however, this difference did not reach statistical significance (log-rank test p = 0.62 for all MACE and p = 0.11 for NC-MACE). The cumulative incidence functions of NC-MACE for both groups are presented in Supplemental Fig. 1A (All MACE) and 1B (NC-MACE). The estimated cumulative incidence of NC-MACE was 11.4% in men with a maxLCBI_4mm_ > 400 vs. 5.4% in men with a maxLCBI_4mm_ < 400 (log-rank test p = 0.003). The estimated cumulative incidence of NC-MACE in women with a maxLCBI_4mm_ > 400 was 15.1% vs. 11.4% in women with a maxLCBI_4mm_ ≤ 400 (log-rank test p = 0.012). The Kaplan-Meier curves of the estimated cumulative incidence of NC-MACE in women and men with the maxLCBI_4mm_ as a predictor are presented in Fig. 1. No differences were found in the cumulative incidences between men and women in both LCBI > 400 (log-rank test p = 0.24, Supplemental Fig. 2A) and LCBI ≤ 400 groups (log-rank test p = 0.43, Supplemental Fig. 2B). At the patient level, maxLCBI_4mm_, operationalized as > 400 vs ≤ 400, significantly predicted future NC-MACE at 24 months for both men and women. The unadjusted HR of a maxLCBI_4mm_ > 400 was 2.10 (95% confidence interval [CI]: 1.28–3.44; p = 0.003) for men and 2.24 for women (95% CI: 1.18–4.30; p = 0.014). There was no significant interaction between maxLCBI_4mm_ > 400 and sex (p = 0.87). The maxLCBI_4mm_ as a continuous variable was also found to be predictive of the occurrence of NC-MACE in both sexes. When the covariates of age, diabetes mellitus, hypertension, chronic renal insufficiency, prior PCI, and ACS were added to the Cox proportional-hazards model, there was no interaction between sex, either with maxLCBI_4mm_ as a binary variable (p = 0.54) or as a continuous variable (p = 0.72).

### 3.3 Endpoints at the plaque level

The overall cumulative incidence of NC-MACE at 24 months as estimated by the Kaplan-Meier method was slightly higher in women than in men at the plaque level (1.5% vs. 0.9%, log-rank test p < 0.0001). The estimated cumulative incidence of NC-MACE was 2.5% in men with a maxLCBI_4mm_ > 400 vs. 0.7% in men with a maxLCBI_4mm_ ≤ 400 (log-rank test p<0.0001). The estimated cumulative incidence of NC-MACE in women with a maxLCBI_4mm_ > 400 was 4.6% vs. 0.9% in women with a maxLCBI_4mm_ ≤ 400 (log-rank test p < 0.001). The Kaplan-Meier curves of the estimated cumulative incidence of NC-MACE with the maxLCBI_4mm_ as a predictor are presented in Supplemental Fig. 3A (men) and 3B (women). At the plaque level, maxLCBI_4mm_ of a Ware segment, operationalized as > 400 vs. ≤ 400, significantly predicted future NC-MACE in the same Ware segment at 24 months for both men and women. MaxLCBI_4mm_ as a binary variable, again using 400 as the cutoff value, was found to be of equal predictive value for both sexes. The HR for men was 3.49 (95% CI: 1.60–7.60; p = 0.002), and the HR for women was 4.79 (95% CI: 2.02–11.38; p < 0.001). Importantly, there was no interaction of maxLCBI_4mm_ as a binary variable and sex (p = 0.57). Similar results were found for maxLCBI_4mm_ as a continuous variable. For men, the HR was 1.43 (95% CI: 1.22–1.67, p < 0.001), and for women, the HR was 1.48 (95% CI: 1.21–1.80, p < 0.001) per 100-unit increase of LCBI.

Of note, the predictive value of maxLCBI_4mm_ as a binary or continuous variable remained high for both men and women, even when adjusted for a PB > 70% and for an MLA ≤ 4 mm^2^. The correlation of the maxLCBI_4mm_ at the plaque level and PB was 0.54 using the Pearson correlation coefficient.

Regarding vulnerable patient-level and vulnerable plaque-level endpoints, the independent correlates of NC-MACE during follow-up are shown in Table 3.

**Table 3:** Primary endpoints unadjusted and adjusted Cox proportional hazards models.

	**MaxLCBI_4mm_ > 400**	**MaxLCBI_4mm_ continuous**
**Vulnerable patient-level models**		
Unadjusted LCBI alone*		
Male	2.10 (1.28–3.44)	1.19 (1.04–1.37)
Female	2.24 (1.18–4.28)	1.25 (1.04–1.49)
*Multivariable adjusted model*†		
Male	2.02 (1.22–3.34)	1.18 (1.03–1.36)
Female	1.54 (0.78–3.07)	1.13 (0.94–1.37)
*Adjusted for variables*		
Age	0.93 (0.76–1.13)	0.93 (0.76–1.14)
Diabetes	1.28 (0.84–1.95)	1.29 (0.84–1.96)
Hypertension	2.12 (1.04–4.31)	2.13 (1.04–4.34)
Chronic renal insufficiency	2.07 (1.18–3.63)	2.04 (1.16–3.58)
History of smoking	1.46 (0.97–2.21)	1.45 (0.96–2.19)
Prior PCI	1.44 (0.96–2.16)	1.46 (0.97–2.18)
ACS	1.23 (0.73–2.08)	1.21 (0.72–2.05)
**Vulnerable plaque-level models**‡		
*Unadjusted LCBI alone*§		
Male	3.49 (1.60–7.60)	1.43 (1.22–1.67)
Female	4.79 (2.02–11.38)	1.48 (1.21–1.80)
*Multivariable adjusted model*‖		
Male	3.02 (1.36–6.71)	1.38 (1.18–1.62)
Female	3.69 (1.50–9.08)	1.39 (1.14–1.70)
*Adjusted for variables*		
Plaque burden > 70% at site of maxLCBI_4mm_	4.11 (1.40–12.06)	2.79 (0.91–8.56)
MLA ≤ 4 mm^2^ at site of maxLCBI_4mm_	1.73 (0.98–3.05)	1.80 (1.02–3.18)

Data are hazard ratio (95% CI). ACS, acute coronary syndrome; maxLCBI_4mm_, maximum 4-mm Lipid Core Burden Index; PCI, percutaneous coronary intervention; MLA, minimum lumen area.

* Interaction between maxLCBI_4mm_ > 400 and sex, p = 0.87. Interaction between maxLCBI_4mm_ continuous and sex, p = 0.70.

† Interaction between maxLCBI_4mm_ > 400 and sex, p = 0.54. Interaction between maxLCBI_4mm_ continuous and sex, p = 0.72.

‡ Patient cluster adjusted via Wei Lin Weissfeld method.

§ Interaction between maxLCBI_4mm_ > 400 and sex, p = 0.57. Interaction between maxLCBI_4mm_ continuous and sex, p = 0.77.

‖ Interaction between maxLCBI_4mm_ > 400 and sex, p = 0.71. Interaction between maxLCBI_4mm_ continuous and sex, p = 0.93.

## 4 Discussion

In this sub-analysis of the LRP study, we investigated whether there was a difference in the ability of maxLCBI_4mm_ detected by NIRS-IVUS to predict future cardiovascular events for both men and women. First, overall cumulative incidence of NC-MACE was not different between men and women, with women having a slightly, non-significantly higher frequency of NC-MACE. Second, the maxLCBI_4mm_ predicted these future NC-MACE for both men and women. The unadjusted HR of a maxLCBI_4mm_ > 400 conveyed a more than twofold risk of future NC-MACE for both sexes. There was no interaction between the maxLCBI_4mm_ and sex, indicating that the maxLCBI_4mm_ was a valid predictor for future NC-MACE irrespective of sex. The absence of interaction between the maxLCBI_4mm_ and sex persisted when the models were further adjusted for other covariates that were significantly different at baseline (Table 3).

In this large dataset of patients with obstructive CAD, women were older and had a higher baseline cardiovascular risk profile than men. This was in line with expectations, as the concept that women have a cardiovascular risk profile distinct from that of men is well-appreciated today [19]. Worse cardiovascular outcomes in women might be anticipated because of this higher risk profile. Some smaller studies reported worse cardiovascular outcomes for women after myocardial infarction [3], [4]. In our study, there was no significant difference in cardiovascular outcomes between men and women at the patient level (defined as the estimated cumulative incidence of both all MACE and NC-MACE at 24 months). At the plaque level, women did have a slightly higher estimated cumulative NC-MACE rate (p < 0.001). The actual NC-MACE rate was very low in both groups, however. The paradox of higher cardiovascular risk but more or less equal cardiovascular outcomes could be explained by several factors, such as a variability in relative risk weighting [19]. Conversely, the sex-related difference of non-obstructive plaque composition, measured by NIRS-IVUS, could be an explanation. A large, prospective imaging study, PROSPECT, demonstrated that vulnerable-plaque characteristics were highly predictive of future non-culprit cardiovascular events [20]. Previous studies demonstrated that despite a higher cardiovascular risk profile, women often have similar, or even more favorable, plaque characteristics in comparison with men as measured by IVUS. In a sex-based sub-analysis of PROSPECT, women had less angiographic stenosis, similar plaque burden, less necrotic core, and less plaque rupture [10]. A sub-analysis of the combined results of the IBIS-3 [21] and Atheroremo-IVUS [22] studies found a smaller plaque burden in women. In both studies, NIRS was also used to detect lipid-rich plaque. There was no baseline difference in the maxLCBI_4mm_ between men and women [5]. Bharadwaj et al. retrospectively analyzed data from 383 patients with stable CAD who underwent clinically indicated angiography and optical coherence tomography (OCT) with NIRS-IVUS in 128 patients. There were no differences in OCT-derived plaque morphology between men and women (maximum lipid arc, lipid length, lipid volume index, minimum cap thickness, incidence of thin-cap fibroatheroma [TCFA], micro-vessels, macrophages, and calcification). The NIRS-derived maxLCBI_4mm_ was also similar [6]. Kataoka et al. imaged non-culprit plaques with OCT in patients with stable angina pectoris (SAP) and ACS. Both in SAP and ACS patients, women had a smaller lipid arc and less calcification and cholesterol crystals, but a similar frequency of TCFA. It was observed that TCFAs in men were clustered more proximally in the arterial segments. Plaque erosion was more prevalent in women with SAP or ACS than in men [9]. In our larger study group, men had more conventional high-risk plaque characteristics as assessed by IVUS. Men had a greater PB than women. Men had more plaque volume and plaque area, even when corrected for BSA. Conversely, the maxLCBI_4mm_ at the plaque level was higher in women. This might suggest that men have more high-risk plaque characteristics as assessed by IVUS but that women have more lipid core as assessed by NIRS. Paradoxically, the maxLCBI_4mm_ was similar between sexes at the patient level. We hypothesize that the group size at the patient level was not sufficient to detect the small differences in maxLCBI_4mm_ between sexes. At the plaque level, there were multiple Ware segments per patient with a corresponding maxLCBI_4mm_ value, thereby creating a larger group size. It is challenging to compare our study results with the aforementioned studies, which were limited by either their retrospective design or small sample size. Thus, the clinical and prognostic relevance of these small differences in plaque composition remain to be assessed.

The high predictive value of NIRS for both sexes in this LRP sub-analysis is further supported by the PROSPECT II study results. PROSPECT II demonstrated that a maxLCBI_4mm_ ≥ 324.7 was independently related to NC-MACE at the patient and plaque levels. Patient-level odds ratio (OR) was 2.27 (95% CI: 1.25–4.13; p = 0.0071) and plaque level OR was 3.80 (95% CI: 1.87–7.70; p = 0.0002) [23]. Likewise, in our study, the predictive value of maxLCBI_4mm_ remained high, even when adjusted for high plaque burden and small lumen area. This indicates that the LCBI can be a good independent predictor of NC-MACE in the same Ware segment, even when corrected for these variables. Theoretically, lipid-rich plaque could be located at a site where there is a small plaque volume or PB but where the lipid core still increases risk. Taken together with our results, the current and previously reported findings suggest that high-risk plaque identification and treatment could be even more important in women, who generally have less severe angiographic stenosis but equal risk for adverse cardiovascular outcomes [10].

### 4.1 Study limitations

The original LRP study was not powered to assess sex differences. Follow-up time was limited to 24 months, which restricted the possible number of non-culprit-related events. A longer follow-up time could have led to more events and a stronger association between the maxLCBI_4mm_ and NC-MACE in both men and women. Non-traditional risk factors, including inflammatory markers or reproductive sex hormones, which are thought to influence cardiovascular risk in women especially, were not collected.

## 5 Conclusion

This sub-analysis of the LRP study showed that the maxLCBI_4mm_ was of similar predictive value for NC-MACE within 24 months in both men and women. NIRS as an addition to IVUS is a valuable diagnostic tool in both women and men to identify NC plaques that are at high risk of causing a subsequent event, either through high PB or high lipid content.

## Authors’ declaration

All authors take responsibility for all aspects of the reliability and freedom from bias of the data presented and their discussed interpretation.

## Clinical Trial Registration

The Lipid-Rich Plaque Study (LRP), https://clinicaltrials.gov/ct2/show/NCT02033694, NCT02033694

## Sources of Funding

The LRP study was sponsored by Infraredx-Nipro, Bedford, MA, USA.

## Declaration of Competing Interests

Ron Waksman, Carlo Di Mario, Hector Garcia-Garcia, Rebecca Torguson were Principal Investigator, European Principal Investigator, Responsible Officer Core Laboratory NIRS-IVUS and angiographic analysis, Worldwide Study Coordinator of the Lipid Rich Trial, sponsored by Infraredx-Nipro, Burlington, MA, USA.

Carlo Di Mario: Grant to Institution: AMGEN, Behring, Chiesi, Daiichi Sanyo, Edwards, Medtronic, Shockwave Medical; Speakers’ fees: Philips.

Ziad Ali: Grants: Abbott Vascular, grants and personal fees: Cardiovascular Systems Inc, personal fees: Amgen, Astra Zeneca, Boston Scientific, other from Shockwave Medical, outside the submitted work.

Hector Garcia-Garcia: Grant to Institution: Medtronic, Biotronik, Neovasc, Boston Scientific, Abbott, Shockwave, Chiesi and Phillips.

Gary Mintz: Honoraria: Boston Scientific, Philips, Medtronic, and Abiomed.

Priti Shah: Employee: Infraredx.

Ron Waksman: Advisory Board: Abbott Vascular, Boston Scientific, Medtronic, Philips IGT, Pi-Cardia Ltd.; Consultant: Abbott Vascular, Biotronik, Boston Scientific, Cordis, Medtronic, Philips IGT, Pi-Cardia Ltd., Swiss Interventional Systems/SIS Medical AG, Transmural Systems Inc., Venous MedTech; Grant Support: AstraZeneca, Biotronik, Boston Scientific, Chiesi, Medtronic, Philips IGT; Speakers Bureau: AstraZeneca; Investor: MedAlliance, Transmural Systems Inc.

All other authors report no relevant disclosures.
