# Supplementary material for: Near-infrared spectroscopy predicts events in men and women: Results from the Lipid Rich Plaque study
Source: Int J Cardiol Heart Vasc. 2022 Mar 8;39:100985. doi: 10.1016/j.ijcha.2022.100985 (PMC8914327; doi:10.1016/j.ijcha.2022.100985)
Supplement: Supplementary data 1 [file mmc1.docx]

**SUPPLEMENTAL MATERIAL**

**Supplemental Figure Legends**

**Supplemental Figure 1A.** Kaplan-Meier curves of the estimated cumulative incidences of All MACE in men and women.
The cumulative incidence of All MACE in women was 18.0% vs. 17.1% in men (log-rank test p=0.62). MACE, major adverse cardiac events. **Supplemental Figure 1B.** Kaplan-Meier curves of the estimated cumulative incidences of NC-MACE in men and women.
The cumulative incidence of NC-MACE in women was 10.3% vs. 7.6% in men (log-rank test p=0.11). NC-MACE, non-culprit major adverse cardiac events.

**Supplemental Figure 2A.** Kaplan-Meier curves of the cumulative incidences of NC-MACE in men vs. women with a maxLCBI_4mm_ >400.

The cumulative incidence of NC-MACE in the LCBI>400 group is 11.4% in men vs. 15.1% in women (log-rank test p=0.24). LCBI, Lipid Core Burden Index; NC-MACE, non-culprit major adverse cardiac events.

**Supplemental Figure 2B.** Kaplan-Meier curves of the cumulative incidences of NC-MACE in men vs. women with maxLCBI_4mm_ ≤400.

The cumulative incidence of NC-MACE in the LCBI<400 group is 5.4% in men vs. 11.4% in women (log-rank test p=0.43). LCBI, Lipid Core Burden Index; NC-MACE, non-culprit major adverse cardiac events.

**Supplemental Figure 3A.** Kaplan-Meier curves of the cumulative incidences of NC-MACE at the plaque level in men with maxLCBI_4mm_ >400 vs. maxLCBI_4mm_ ≤400.

In men, the cumulative incidence of NC-MACE at the plaque level in the LCBI>400 group is 2.5% vs. 0.7% in the LCBI ≤400 group (log-rank test p=<0.0001). LCBI, Lipid Core Burden Index; NC-MACE, non-culprit major adverse cardiac events.

**Supplemental Figure 3B.** Kaplan-Meier curves of the cumulative incidences of NC-MACE at the plaque level in women with maxLCBI_4mm_ >400 vs. maxLCBI_4mm_ ≤400.

In women, the cumulative incidence of NC-MACE at the plaque level in the LCBI>400 group is 4.6% vs. 0.9% in the LCBI ≤400 group (log-rank test p<0001). LCBI, Lipid Core Burden Index; NC-MACE, non-culprit major adverse cardiac events.

**Supplemental Figure 1A**

**Supplemental figure 1B**

**Supplemental Figure 2A**

**
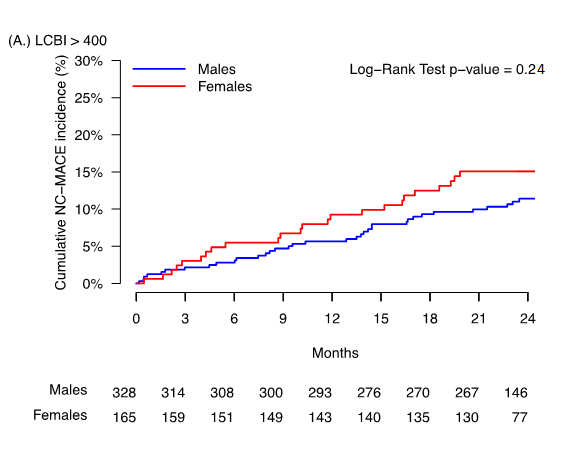
**

**Supplemental Figure 2B**

**Supplemental figure 3A**

**Supplemental figure 3B**

**Supplemental Table 1.** Kaplan‐Meier estimates for cumulative rates of patient‐level non‐ culprit MACE at 2 Years

| **NC-MACE component** | **Percent (number of patients)** |
| --- | --- |
| Cardiac Death | 2 (24) |
| Cardiac Arrest | 0.3 (4) |
| ACS | 4 (47) |
| Non‐Fatal MI | 3 (35) |
| Rehospitalization with Progressive Angina  and >20% Disease  Progression | 0.7 (8) |
| Revascularization | 6 (69) |
| PCI | 5 (57) |
| CABG | 1 (13) |

Data are percent (number). NC-MACE, non-culprit major adverse cardiac events; ACS, acute coronary syndrome; MI, myocardial infarction; PCI, percutaneous intervention; CABG, coronary artery bypass grafting.
